# Variations and Challenges of Cutaneous Oncological Surgery in Morocco

**DOI:** 10.7759/cureus.106741

**Published:** 2026-04-09

**Authors:** Israe El Ghazouli, Salim Gallouj, Ouiame El Jouari

**Affiliations:** 1 Dermatology Department, Mohammed VI University Hospital, Faculty of Medicine and Pharmacy, Abdelmalek Essaâdi University, Tangier, MAR

**Keywords:** dermatologic surgery, guidelines, oncological skin surgery, patient management, ­skin cancer, wide local excision (wle)

## Abstract

Background

Oncological skin surgery is a cornerstone in the management of malignant skin tumors; however, no national or international guidelines exist regarding surgical techniques.

Objectives

This study aimed to assess variation in oncological skin surgery practices and to explore the influence of clinicians’ specialty and professional grade on this variation.

Methods

This national, anonymized, cross-sectional study used an online questionnaire distributed to dermatologists, plastic and reconstructive surgeons, and other specialists performing oncological skin surgery. Data were analyzed using SPSS version 27 (IBM Corp., Armonk, NY, USA). Categorical variables were summarized as frequencies and percentages. A p-value < 0.05 was considered statistically significant.

Results

Among the participants, 81.9% were dermatologists and 11.4% were plastic surgeons. The remaining 6.7% were distributed across other specialties, including maxillofacial surgery (2.6%), general surgery (2.0%), and breast surgery (2.1%); 61.4% were consultants. Most clinicians reported learning surgical techniques informally from senior colleagues (92.1%). Significant variation was observed in wide local excision (WLE) planning and execution. For a 1 cm WLE, 65.9% marked margins from the scar edge, whereas plastic surgeons more frequently measured from the scar center than dermatologists (p = 0.003). Most excisions followed a longitudinal or oblique limb axis (61.4%), and only 40% of clinicians sent dog ears for histological examination. Excision depth varied, most commonly extending to the deep fascia (53.5%). Skin cancers were excised without prior biopsy in 71.3% of cases, with one-stage repair performed in 92.2%, most often by direct closure (83.4%). Complete excision was achieved in 95.2% of cases, with excellent clinicopathological correlation in 89.5%. The overall complication rate was low (3%) but was significantly higher in patients receiving antithrombotic therapy (p < 0.05).

Conclusion

Substantial variation exists in oncological skin surgery practices. The development of standardized consensus guidelines may improve procedural consistency and potentially enhance patient outcomes.

## Introduction

Skin cancers are the most common malignancies in adults. Their incidence is steadily increasing. This rise is linked to longer life expectancy and changes in behavior, especially repeated sun exposure. Surgical excision remains the first-line treatment [1].

In Morocco, malignant skin tumors are a significant public health concern. Diagnosis is often delayed. Management can be complex and costly. To date, few national studies have specifically addressed malignant skin tumors in this context [2].

The practice of dermatologic surgery has expanded significantly and become increasingly structured in recent years, particularly in the management of skin cancers. The exponential rise in their incidence, partly attributable to population aging, together with the increasing incidence of melanoma, has led to a growing demand for dermatologic surgical procedures [3].

A thorough understanding of these tumors is essential for optimal management, as their specific anatomical and clinical characteristics largely determine prognosis. Dermatologists play a central role in initial patient management, as they are qualified to establish a clinical diagnosis, determine the appropriate surgical approach, and perform the procedure (either a diagnostic biopsy or immediate excision). They are also responsible for discussing histopathological findings and determining the need for additional treatment, particularly with regard to surgical follow-up. In this context, evidence-based recommendations clearly guide the management of melanoma, as well as basal cell carcinoma (BCC) and squamous cell carcinoma (SCC) [4-6].

Although plastic surgeons are increasingly consulted for skin tumor management, their availability for superficial cutaneous surgery has decreased, further reinforcing the role of dermatologists in this field. To our knowledge, no prospective studies have previously reported data on the surgical excision of skin tumors performed by dermatologists in Morocco. The aim of this study was therefore to provide both qualitative and quantitative data on dermatologic surgical activity, in order to better characterize the contribution of dermatologists to the overall care pathway and to assess whether variations in surgical practice among specialties may influence patient outcomes.

## Materials and methods

Study design and setting

This was a national, cross-sectional observational study conducted over a six-month period, from May 2025 to October 2025, and reported in accordance with the STROBE (Strengthening the Reporting of Observational Studies in Epidemiology) guidelines. Data were collected using a structured, anonymous online questionnaire (Appendix).

Participants

Eligible participants were physicians involved in the surgical management of skin tumors who routinely perform wide local excision (WLE). Survey invitations were disseminated through national professional associations representing specialties involved in oncologic and dermatologic surgery, including dermatology, plastic surgery, and other surgical disciplines. Participation was voluntary and anonymous.

Data sources and variables

Data were collected via a standardized online questionnaire hosted on Google Forms (Google, Mountain View, CA, USA). The questionnaire captured information on demographic characteristics (specialty, professional status, and years of practice); training background in WLE; surgical practices, including incision orientation, excision depth, margin determination, and reconstruction techniques; preoperative practices, such as skin marking and use of local anesthesia; management of excision margins, including consideration of margins from prior biopsies; histopathological practices; and postoperative outcomes and complications.

Participants were asked to describe their routine approach to planning and performing WLE, including perioperative decision-making and complication management. For questions related to learning sources and complications, multiple responses were permitted, allowing respondents to select more than one applicable option.

Bias

Selection bias may have occurred due to the voluntary nature of participation and the use of professional association mailing lists. To minimize information bias, responses were anonymized, and standardized questions were used.

Statistical analysis

Data were coded and analyzed using SPSS software (version 27; IBM Corp., Armonk, NY, USA). Descriptive statistics were used to summarize participant characteristics and surgical practices. Categorical variables were presented as frequencies and percentages, while continuous variables were expressed as means with standard deviations or medians with interquartile ranges, depending on data distribution.

Comparisons between groups (e.g., dermatologists vs. plastic surgeons and consultants vs. registrars) were performed using the Chi-squared test for categorical variables when expected cell counts were adequate. Fisher’s exact test was applied when expected frequencies were <5. For continuous variables, Student’s t-test was used when normality assumptions were met; otherwise, non-parametric tests were applied.

For each statistically significant result, the corresponding test statistic (χ² or Fisher’s exact test) and p-value were recorded. All statistical tests were two-tailed, and a p-value < 0.05 was considered statistically significant.

Incomplete responses were not imputed. Analyses were conducted using available data for each variable, and percentages were calculated using item-specific denominators. The total sample size (n = 156) refers to eligible respondents included in the final analysis after exclusion of ineligible or duplicate entries. All statistical tests were two-tailed, and a p-value < 0.05 was considered statistically significant.

## Results

Study population

A total of 156 responses were included in the final analysis. Percentages are reported using item-specific denominators, as some survey items allowed multiple responses or had missing data.

Regarding specialty, 127 participants (81.4%) were dermatologists, 21 (13.5%) were plastic surgeons, and eight (5.1%) belonged to other surgical specialties (including maxillofacial, general, and breast surgery). Most respondents were consultants (n = 97; 62.2%), while 48 (30.8%) were registrars or specialist registrars, and 11 (7.0%) belonged to other professional categories.

Training and learning sources for WLE

Almost all respondents (n = 144; 92.3%) reported learning WLE techniques from senior consultants or professors. Multiple responses were permitted for this item. Learning sources included consultant dermatologists (n = 81; 51.9%), consultant plastic surgeons (n = 76; 48.7%), and consultants from other specialties (n = 22; 14.1%). Additional resources included textbooks (n = 76; 48.7%) and online instructional videos (n = 76; 48.7%), explaining why cumulative percentages exceeded 100%.

Surgical planning and incision technique

**Figure 1:**
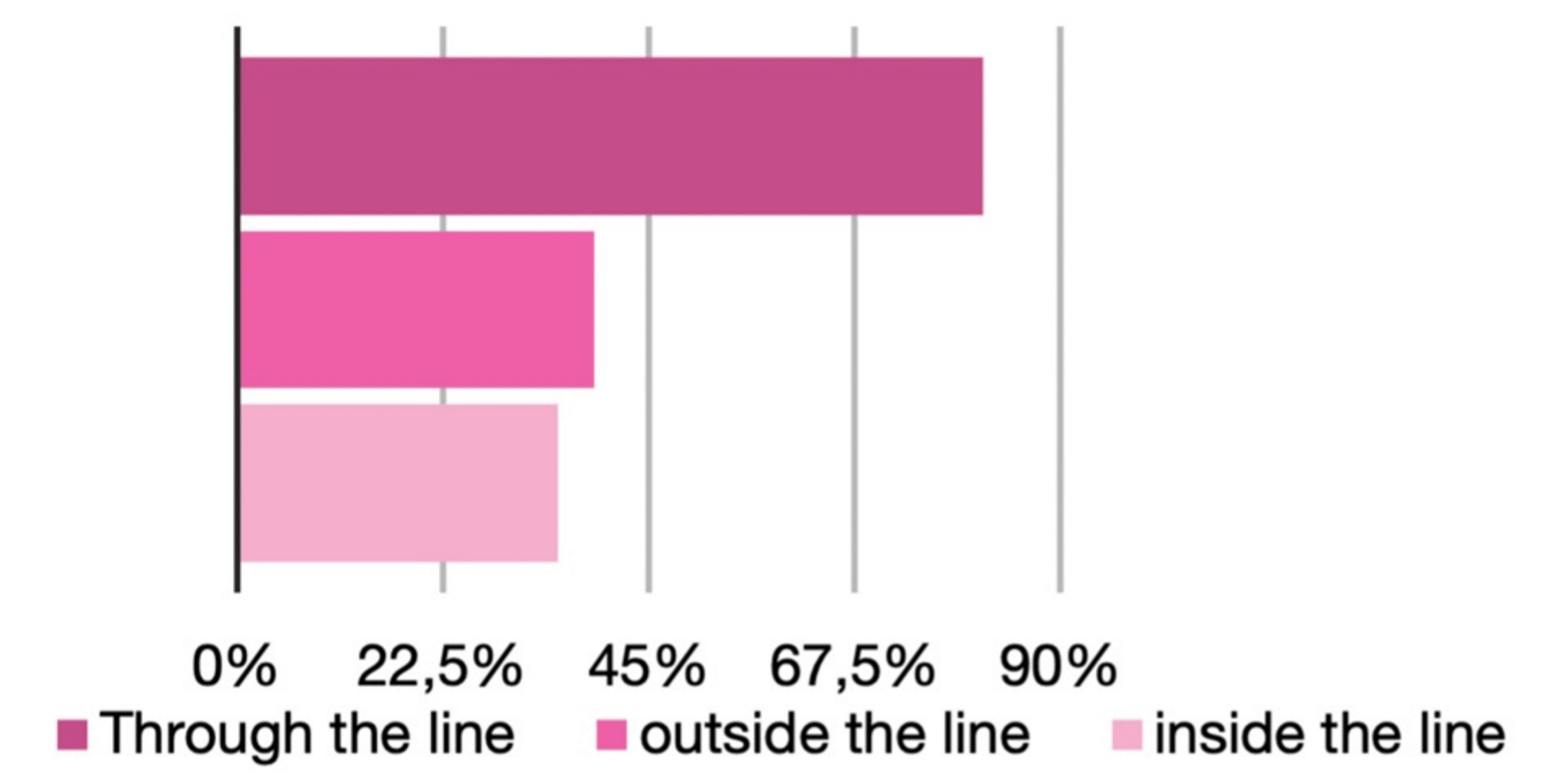
Incision line

Variation was observed in incision orientation. Most respondents (n = 94; 60.3%) oriented WLE incisions longitudinally or obliquely, 56 (35.9%) transversely, and six (3.8%) reported alternative orientations. After skin marking, 81 respondents (52.3%) incised directly through the marked line, 39 (25.2%) outside the line, and 35 (22.6%) inside the line (Figure 1).

**Figure 2:**
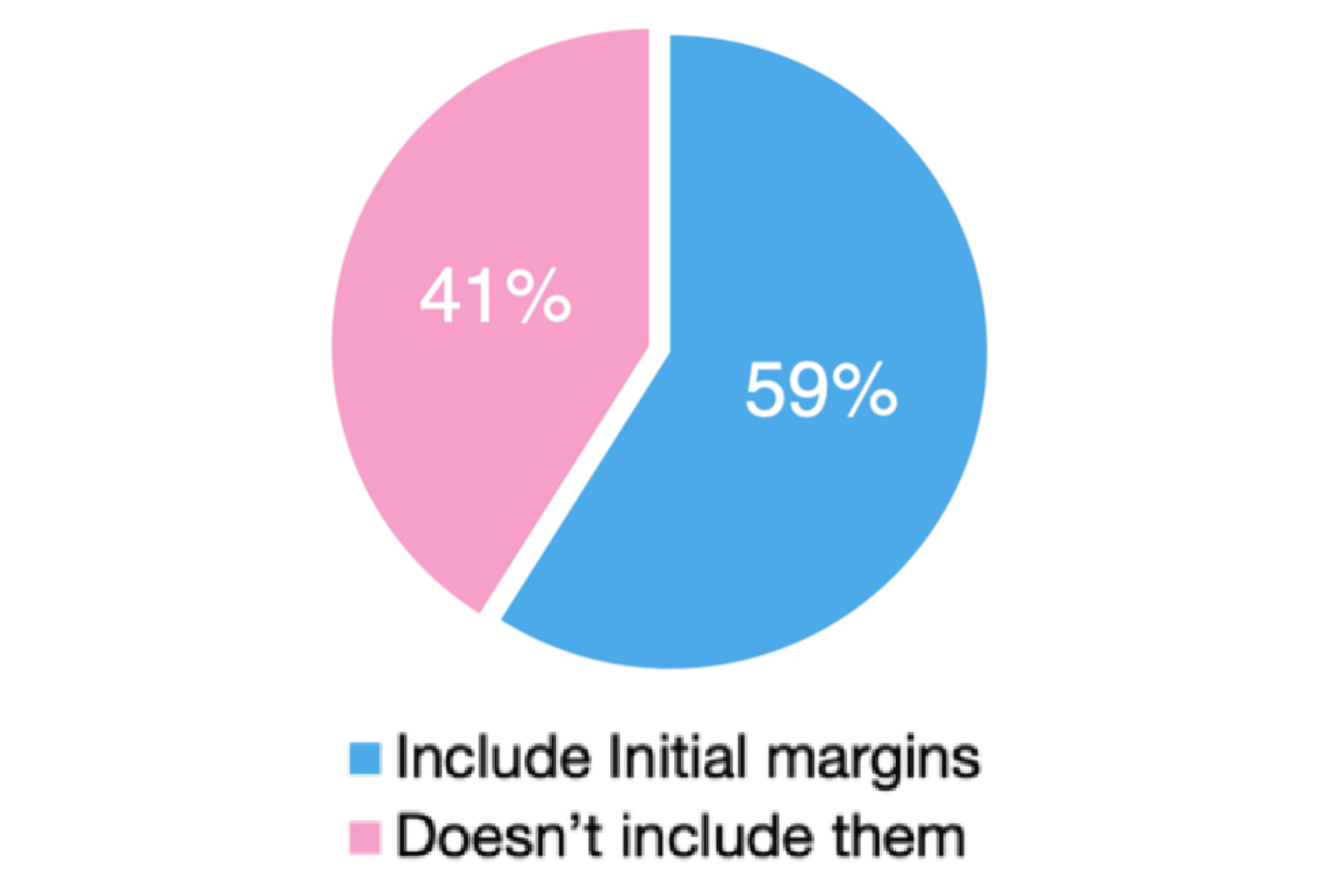
Surgical revision

**Figure 3:**
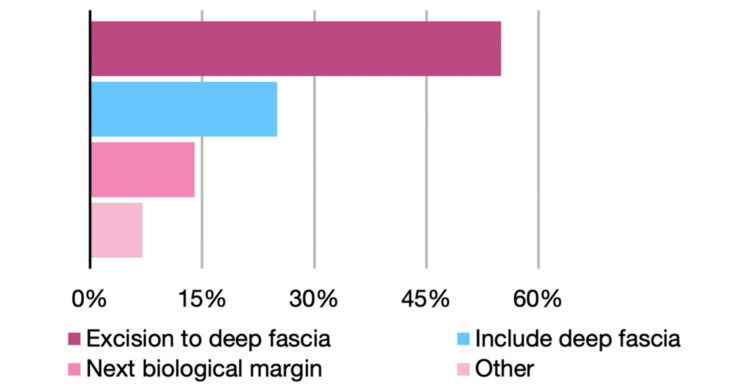
Depth of excision

Participants were divided on whether to incorporate margins from the initial narrow excision, with 92 respondents (59.0%) including these margins and 64 (41.0%) excluding them (Figure 2). Regarding excision depth, 85 respondents (54.5%) excised down to the deep fascia, 31 (19.9%) to the next biological plane, and five (3.2%) included the deep fascia itself (Figure 3).

Margin measurement and reconstruction practices

**Figure 4:**
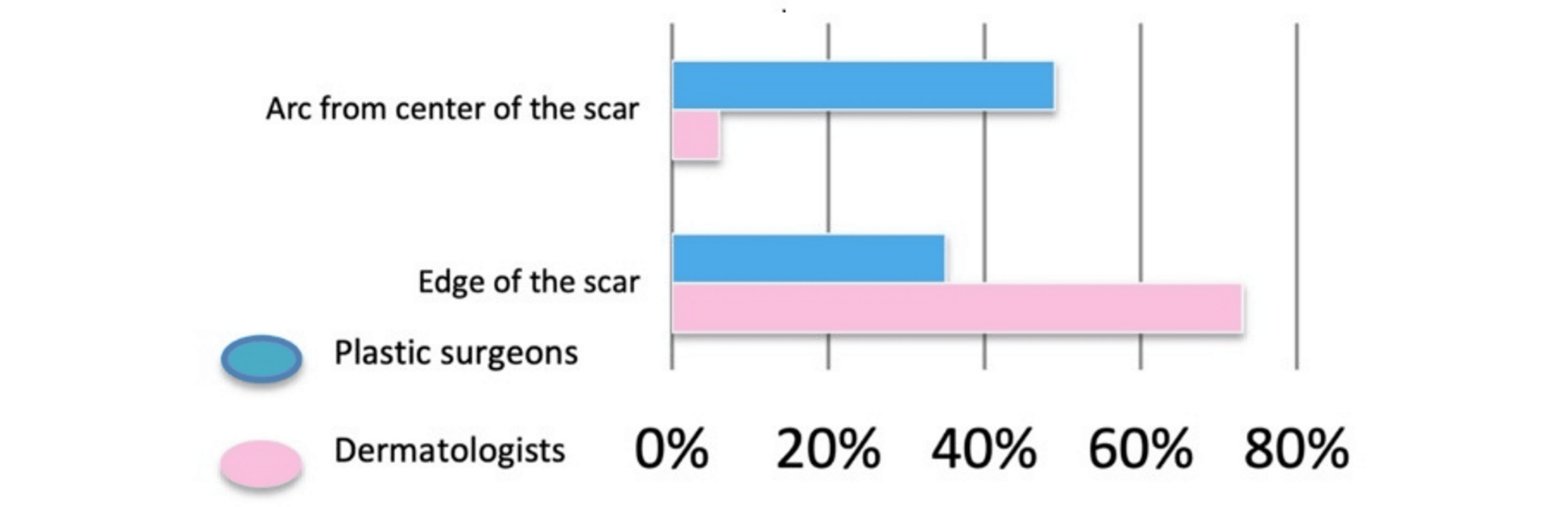
Calculation of the margin arc

Most respondents (n = 118; 75.6%) measured margins from the edge of the biopsy scar, while 25 (16.0%) measured from the central portion of the scar. Plastic surgeons were significantly more likely than dermatologists to measure margins from the center of the scar (29% vs. 8%), whereas dermatologists more frequently measured from the scar edge (92% vs. 71%) (Chi-squared test, χ² = 8.84; p = 0.003) (Figure 4).

When dog-ear correction was performed, 94 respondents (60.3%) did not submit the excised tissue for histopathological examination (Fisher’s exact test; p = 0.048).

**Figure 5:**
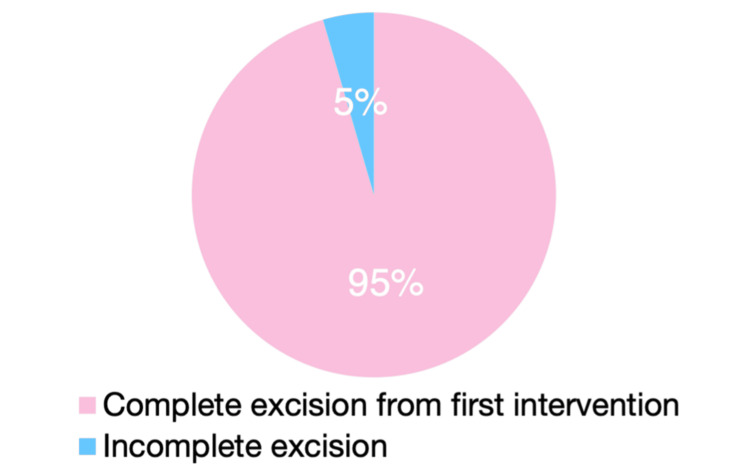
Complete excision

Reconstruction was completed in a single operative stage in 144 cases (92.3%), with direct closure being the most commonly used technique (n = 130; 83.3%) (Chi-squared test; p = 0.03) (Figure 5).

Excision outcomes and complications

**Figure 6:**
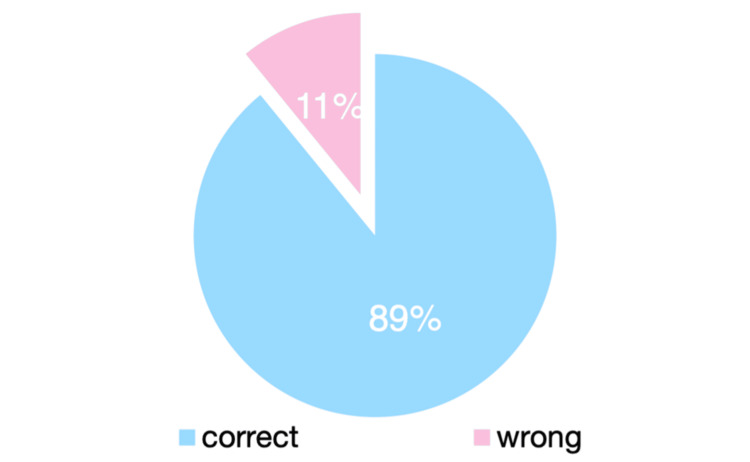
Clinicopathological correlation

Complete excision at the initial procedure was achieved in 148 cases (95.2%), and clinicopathological concordance was reported in 139 cases (89.5%) (Chi-squared test; p = 0.027) (Figure 6).

The overall complication rate was 3.0%, calculated on an event-based basis rather than per patient, as respondents could report multiple complication types for the same patient. Therefore, the total number of reported complication events exceeded the number of affected patients. Reported complications included hematoma (n = 16), suture dehiscence (n = 16), edema (n = 13), infection (n = 11), vasovagal episodes (n = 8), and tissue necrosis (n = 3).

Complication events were significantly more frequent among patients receiving antithrombotic therapy at the time of surgery compared with those not receiving antithrombotic treatment (Fisher’s exact test; p < 0.05), although absolute complication rates remained low (Tables 1, 2).

**Table 1:** Distribution of reported complication events

Complication type	Number of events (n)
Hematoma	16
Suture dehiscence	16
Edema	13
Infection	11
Vasovagal episodes	8
Tissue necrosis	3
Total	67

**Table 2:** Summary of complication analysis

Parameter	Value/description
Overall complication rate	3.0% (event-based)
Method of calculation	Multiple events per patient allowed
Patients on antithrombotic therapy	Higher complication frequency
Patients without therapy	Lower complication frequency
Statistical test	Fisher’s exact test
Significance	p < 0.05
Interpretation	Statistically significant difference; absolute rates remained low

Complication rate was calculated on an event-based basis rather than per patient, as multiple complications could occur in a single patient.

## Discussion

This study highlights the significant involvement of dermatologists in the surgical management of skin tumors. It also reflects the substantial role of surgery within cutaneous oncology, as the management of skin tumors frequently necessitates surgical intervention.

Considerable variation was observed in how surgery was planned and performed among respondents. Most clinicians reported acquiring skills in WLE primarily through mentorship and guidance from colleagues, with limited reliance on formal educational resources such as textbooks or instructional videos. The only statistically significant difference identified between dermatologists and plastic surgeons concerned the method used to calculate the surgical margin arc: dermatologists more often measured margins from the edge of the biopsy scar, whereas plastic surgeons tended to measure from the central 50% of the scar. Variability was also noted in whether clinicians accounted for the peripheral and deep margins already obtained during the initial narrow-margin excision. Although the 2022 NICE guidelines recommend incorporating these margins, this variability underscores the need for clearer guidelines and more standardized procedures. Specifically, the clinical margin should be measured around the histological biopsy scar while taking into account the recommended primary melanoma margins [7].

Nearly one-third of respondents (29%) reported stretching the skin when measuring margins, a practice that may reduce the effective margin obtained. Only a small proportion (5%) injected local anesthetic before marking the skin, a factor that may also influence margin size. Additionally, significant inconsistency was observed regarding whether incisions were made through, outside, or inside the skin markings, which may further affect final margin outcomes.

There was broad agreement that WLE scars on the limbs should be oriented longitudinally or obliquely. This is particularly important because lymphatic vessels in the upper and lower limbs run predominantly in a vertical direction. Consequently, transverse incisions may damage lymphatic structures, impair lymphatic drainage, and increase the risk of complications such as lymphoedema [8,9].

Analysis of the international literature confirms the predominant role of dermatologists in the surgical management of skin tumors. Manternach et al. conducted a retrospective study in the United States comparing the number of tumors excised and the surgical techniques used by dermatologists, general surgeons, and plastic surgeons in 1998 and 1999. Excluding biopsies, dermatologists treated skin carcinomas 80 times more frequently than general surgeons and seven times more frequently than plastic surgeons. Reconstruction techniques included simple suturing, local flaps, and skin grafts [10].

In Germany, Kunte et al. reported a marked increase in the number of skin tumors surgically treated by dermatologists in a large single-center retrospective study spanning from 1971 to 2006 [11]. In 2006, 4,031 procedures were performed compared with 1,892 in 1971, representing a 113% increase. A total of 101,103 procedures were recorded over the study period. The authors emphasized the importance of comprehensive knowledge of skin tumors to optimize surgical decision-making. Similarly, Hensen et al. documented 164,487 dermatologic surgical procedures performed across 78 academic and non-academic dermatology departments in Germany, encompassing a wide range of interventions from superficial procedures to wide excisions with locoregional flap reconstruction [12].

In Spain, Fernández-Jorge et al. described the dermatologic surgical activity of a single dermatology department during 2003, reporting 644 procedures performed in 565 patients [13]. All procedures were related to malignant tumors, including 240 BCCs, 117 SCCs, and 77 melanomas. As in our study, direct suturing was the most frequently used closure technique (346 cases), followed by flap reconstruction (133 cases). However, this series reported a notably high number of skin grafts (129 cases). As observed in our cohort, the authors emphasized the high accuracy of clinical diagnoses made by dermatologists and a very high rate of complete excision at the initial procedure (92%). They also reported a low complication rate (3%), including suture dehiscence, infection, and hemorrhage. The authors highlighted the clinical and economic importance of dermatologic surgery, noting that outpatient management reduces both hospitalization time and overall healthcare costs associated with skin cancer treatment [14].

Our study further demonstrates, based on a large number of cases, the excellent diagnostic accuracy of dermatologists, with a clinical-histopathological concordance rate of 89.5%. This figure is comparable to rates of 90.8% and 84.4% reported in Spanish studies [15]. This diagnostic expertise enables dermatologists to select excision margins that adhere closely to current guideline recommendations. Surgical margin guidelines vary internationally, ranging from 1 to 3 cm, which may result in excision defects measuring 2 to 6 cm in diameter. Increasing concern has been raised regarding the potentially unnecessary morbidity associated with larger excision defects [7]. The diagnostic efficiency of dermatologists allows for immediate and appropriate surgical management, reducing the need for multiple therapeutic steps and ultimately optimizing healthcare costs [15].

Moreover, several studies have demonstrated that continuation of antithrombotic therapy does not increase the risk of bleeding during dermatologic surgery. In contrast, discontinuation of antithrombotic agents has been associated with an increased risk of perioperative thrombotic events, including stroke and myocardial infarction. Consequently, the benefit-risk balance favors continuation of antithrombotic therapy in dermatologic surgery, provided that perioperative hemostasis is carefully managed, the international normalized ratio (INR) is maintained at or below three, and high-risk patients are closely monitored postoperatively. Detailed recommendations for the management of antithrombotic therapy in dermatologic surgery are available in the literature [16-27].

Limitations

The use of an online questionnaire may have introduced selection bias, potentially leading to underrepresentation of certain specialties or practice settings. Additionally, although national in scope, the sample size may not fully capture all variations in clinical practice. Finally, this study did not evaluate clinical outcomes in relation to specific surgical techniques, limiting conclusions regarding the direct impact of practice variation on patient outcomes.

## Conclusions

To our knowledge, this study constitutes the first national prospective multicenter assessment of dermatologic surgery activity. Although it does not capture the full spectrum of dermatologic surgical practice, given its limited duration and non-exhaustive design, it nonetheless provides a robust and representative overview of current practices. The strength of this work lies in the substantial number of participating centers and practitioners, allowing for meaningful analysis of variations in surgical approaches, resource availability, and case management across different settings. The role of dermatologists in the surgical management of cutaneous tumors has become an increasingly important topic of discussion, particularly in the context of the exponential rise in skin cancer incidence driven by population aging and cumulative ultraviolet exposure. This epidemiological shift places growing demands on dermatologic services and highlights the need for optimized, standardized, and evidence-based surgical care. In this context, our findings provide valuable insights into the real-world challenges faced by dermatologists, including heterogeneity in surgical techniques, disparities in access to specialized equipment, variations in training, and organizational constraints.

There is significant variation in dermatologic oncological surgery practices in Morocco, and understanding these differences can help guide clinical decision-making and identify areas for improvement. By documenting these variations, this study underscores the critical role of dermatologists as primary surgical providers for skin cancer and emphasizes the necessity of strengthening surgical training, harmonizing clinical practices, and improving resource allocation. Ultimately, such efforts are essential to ensure high-quality, safe, and equitable dermatologic surgical care in the face of a rapidly expanding burden of cutaneous malignancies.

## Appendices

Variations and challenges in skin oncologic surgery in Morocco

Thank you for your participation. This questionnaire will take approximately 3 minutes.

*Indicates a required question

1. You are: *

Dermatologist

Plastic surgeon

Other surgical specialties: traumatology, general surgery, maxillofacial

2. Your professional status: *

Specialist

Resident

Professor

3. Your years of experience: *

Less than 5 years

5-10 years

More than 10 years

4. Your practice setting: *

Hospital

Private office/clinic

5. From whom did you learn cutaneous oncologic surgery? *

Professors in plastic surgery/dermatology

Specialist doctors in surgery/dermatology

Books

Online videos

Other

6. Have patients recruited for surgery already received prior topical treatment? *

Yes

No

7. If yes, which treatment?

8. How would you mark the margins to perform a 1 cm wide local excision? *

9. How would you mark your margins for a thickened lesion to achieve a 1 cm wide local excision? *

10. How do you orient incisions for an initial narrow-margin excision on the limbs? *

11. Do you orient your excision according to relaxed skin tension lines (RSTL)? *

Yes

No

12. Regarding local anesthesia (LA) for a wide local excision, do you:

Infiltrate before marking margins

Infiltrate after marking margins

13. Regarding skin marking for a wide local excision, do you:

Mark margins with the skin stretched

Mark margins with the skin relaxed

14. Regarding marking during a surgical re-excision, do you consider the peripheral and deep margins already obtained during the initial excision?

Yes

No

15. Regarding excision depth for a wide local excision, which of the following do you aim to achieve?

Down to the deep fascia

Include the deep fascia

Down to the next biological plane (periosteum, perichondrium, etc.)

16. During a wide local excision, how does your incision relate to the skin markings you have drawn?

Incise inside the line

Incise through the line

Incise outside the line

17. If performing a dog-ear repair, do you send the tissue for histology?

Yes

No

18. In general, is reconstruction performed at the same time as excision?

Yes

No (performed in two stages)

19. Type of reconstruction used:

Local/regional flap

Skin graft

Secondary intention healing

20. Was the initial excision complete?

Yes

No

21. Was the correlation between the clinically suggested diagnosis and histopathological analysis correct?

Yes

No

22. Have most of your patients experienced complications?

Yes

No

23. If yes, which complications?

Hematoma

Suture dehiscence

Infection

Necrosis

Other

24. Are these patients on antithrombotic therapy?

Yes

No

25. Were any of these patients hospitalized following complications?

Yes

No
